# Genetic Algorithm-Enhanced Direct Method in Protein Crystallography

**DOI:** 10.3390/molecules30020288

**Published:** 2025-01-13

**Authors:** Ruijiang Fu, Wu-Pei Su, Hongxing He

**Affiliations:** 1 Department of Physics, School of Physical Science and Technology, Ningbo University, Ningbo 315211, China; 2011077041@nbu.edu.cn; 2 Department of Physics and Texas Center for Superconductivity, University of Houston, Houston, TX 77204, USA; wpsu@uh.edu

**Keywords:** direct method, genetic algorithm, protein crystallography, phase problem, non-crystallographic symmetry, parallel computing

## Abstract

Direct methods based on iterative projection algorithms can determine protein crystal structures directly from X-ray diffraction data without prior structural information. However, traditional direct methods often converge to local minima during electron density iteration, leading to reconstruction failure. Here, we present an enhanced direct method incorporating genetic algorithms for electron density modification in real space. The method features customized selection, crossover, and mutation strategies; premature convergence prevention; and efficient message passing interface (MPI) parallelization. We systematically tested the method on 15 protein structures from different space groups with diffraction resolutions of 1.35∼2.5 Å. The test cases included high-solvent-content structures, high-resolution structures with medium solvent content, and structures with low solvent content and non-crystallographic symmetry (NCS). Results showed that the enhanced method significantly improved success rates from below 30% to nearly 100%, with average phase errors reduced below 40°. The reconstructed electron density maps were of sufficient quality for automated model building. This method provides an effective alternative for solving structures that are difficult to predict accurately by AlphaFold3 or challenging to solve by molecular replacement and experimental phasing methods. The implementation is available on Github.

## 1. Introduction

The accurate determination of three-dimensional protein structures is crucial for understanding their biological functions, facilitating drug design, and advancing life science research. Despite the remarkable progress in protein structure prediction achieved by AlphaFold3 in 2024 [1], experimental methods remain indispensable, particularly for novel sequences and multi-chain complexes where AI predictions may have limited accuracy. High-resolution experimental structures not only provide reliable foundations for structural biology research but also serve as valuable training data for the improvement of AI models.

X-ray crystallography remains the primary experimental technique for determining atomic-resolution structures of macromolecules. While the interaction between X-rays and crystalline electrons produces regular diffraction patterns, detectors can only record diffraction intensities, not phases. This “phase problem” represents the fundamental challenge in X-ray diffraction methods, as phase information is essential for reconstructing electron density maps. Current approaches to the phase problem include both experimental and computational methods. Experimental methods like heavy atom techniques (isomorphous replacement and anomalous dispersion) require preparation of heavy atom derivatives. Computational methods such as molecular replacement (MR) rely on homologous structures (>30% sequence identity) or accurate structure predictions [2,3]. Although AlphaFold3 has expanded the applicability of MR, its predictions may be insufficient for novel structures.

Direct methods require only diffraction amplitude data without additional prior information, making them particularly suitable for solving novel structures while avoiding model bias. The development of direct methods has progressed from small molecules to macromolecules through several key breakthroughs. Early probabilistic approaches based on the Sayre equation, triple phase relationships, and tangent formulas are primarily effective for small molecules [4,5,6,7,8,9]. However, their effectiveness significantly decreases for structures with resolutions lower than 1.2 Å or those containing over 1000 non-hydrogen atoms per asymmetric unit [10]. To overcome this limitation, researchers developed dual-space iterative projection methods, alternating constraints between real and reciprocal space to find optimal solutions satisfying all constraints. Significant advances in this field include Fienup’s hybrid input–output (HIO) algorithm with a negative feedback mechanism [11], histogram matching techniques for protein density modification [12], and various solvent region optimization approaches [13,14]. Recent years have witnessed continued innovation in direct methods. The development of difference map (DM) techniques [15,16,17,18], the extension of HIO algorithms to macromolecular structure determination [19,20,21,22], the utilization of electron density connectivity for protein envelope determination [23,24], and the integration of deep learning approaches have significantly advanced the field [25]. However, traditional direct methods still face limitations in success rate, iteration requirements, and applicability to low-solvent-content crystals [26,27,28,29].

Genetic algorithms (GAs) have demonstrated unique advantages in structural biology by simulating biological evolution’s selection, crossover, and mutation processes for global optimization [30]. Previous attempts to combine genetic algorithms with direct methods have shown promise in crystallography [31,32]. Their applications in crystallography span from determining heavy atom positions [33] and molecular replacement [34] to small-molecule structure reconstruction [35] and extend from powder diffraction data processing [36] to small-angle X-ray scattering analysis [37]. Truong et al. explored a GA-enhanced HIO algorithm for non-periodic structures [38]. Kantamneni et al. systematically studied parameter optimization and fitness function design for phase improvement [39,40]. Despite initial success, these methods face significant limitations. The existing approaches are either restricted to centrosymmetric small molecules at high resolutions [35], require initial phase estimates [40], or focus primarily on phase optimization in reciprocal space without effectively utilizing real-space constraints.

At its core, protein crystal structure determination represents a complex non-convex optimization problem, requiring the search for an optimal solution satisfying multiple constraints within a vast search space. Traditional direct methods apply distinct constraints in protein and solvent regions but often ignore correlations between adjacent grid points. Due to the lack of global optimization guidance and information sharing between independent reconstructions, these inherent algorithmic limitations often lead to local optima or iteration stagnation. Particularly for larger protein structures, even when favorable electron density emerges in local regions, it becomes challenging to extend this into a complete, interpretable density map. Previous attempts to combine genetic algorithms with direct methods have focused primarily on phase optimization in reciprocal space, without effectively utilizing solvent region constraints. Most critically, these approaches have been unable to achieve ab initio determination of protein structures from random phases.

In this study, we propose an innovative strategy that deeply integrates genetic algorithms with direct methods to overcome these limitations. Our approach uniquely leverages the constant density constraint of bulk solvent regions while utilizing population evolution mechanisms to enrich common and complementary information from multiple independently reconstructed electron densities. Through message passing interface (MPI) parallel computing, we simultaneously generate multiple independent electron density reconstructions as the GA population, performing selection, crossover, and mutation operations at fixed iteration intervals. Given this fundamental advancement, our comparative analysis focuses on the performance improvements achieved between traditional direct methods and our GA-enhanced direct methods, as demonstrated by increased success rates, reduced iteration counts, and improved phase accuracy in systematic tests of the same protein structures.

The key innovations of our method include the following: (1) integration of the constant density constraint of bulk solvent regions into a hybrid input–output algorithm as a strong physical constraint, (2) implementation of a population co-evolution strategy in real space that promotes effective phase information iteration, and (3) the ability to perform ab initio structure determination from random phases without requiring initial phase estimates. Through systematic comparison of structure reconstruction effects before and after improvement, we demonstrate significant enhancements in iteration success rates, reduction in required iteration counts, improvements in phasing quality, and acceleration of the phasing process through MPI parallel computing. These improvements make the method particularly valuable for challenging cases such as low-solvent-content structures and those with non-crystallographic symmetry, representing a significant advancement in protein crystallography. However, like traditional direct methods, our GA-enhanced approach still has certain limitations. It generally requires protein crystals with relatively high solvent contents and good-quality diffraction data. For structures with very low solvent content or poor diffraction quality, the method’s effectiveness may be significantly reduced due to insufficient density constraints from the solvent region.

## 2. Materials and Methods

### 2.1. Overview of Genetic Algorithm-Enhanced Direct Method

**Figure 1:**
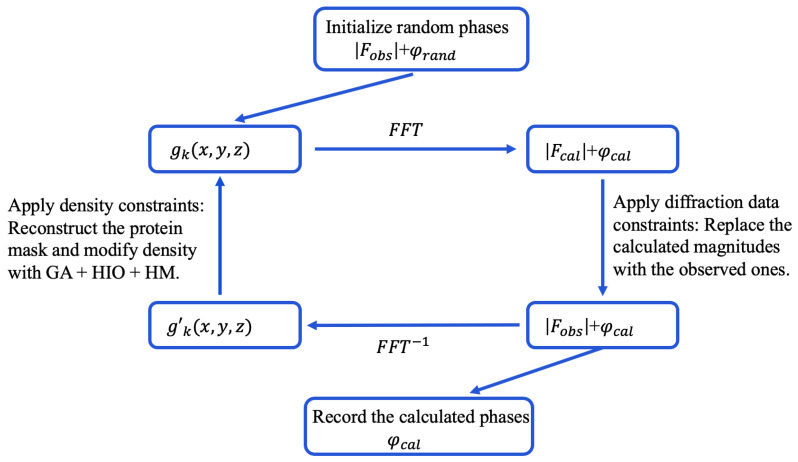
Flowchart of the direct phasing method. Subscript *k* denotes the iteration number. $g_{k}$ is the reconstructed electron density of the protein structure.

Direct methods enable unbiased protein structure determination by iteratively applying dual-space constraints without prior structural information [20,21,26]. The method alternates between reciprocal and real space to obtain electron density distributions satisfying constraints in both spaces. Starting from a random electron density ($g_{k}$) defined in real space, the process converts it to reciprocal space through FFT, yields calculated amplitudes ($|F_{cal}|$) and phases ($\varphi_{cal}$), replaces $|F_{cal}|$ with experimental observations ($|F_{obs}|$), and transforms back to real space to obtain the modified density ($g_{k}^{\prime }$). Figure 1 shows a flowchart of the direct phasing method.

However, traditional direct methods face significant limitations [26,29]. The most critical issue lies in their lack of global optimization guidance. Current algorithms modify each grid point’s electron density without considering spatial correlations, reducing the convergence radius. Although the HIO algorithm’s negative feedback aids in convergence, the independent evolution of densities without information sharing often leads to local minima. A case study (protein with PDB ID 1no4) in Section 3.2.1, clearly demonstrates these limitations. Independent electron density reconstructions from different random initial values, even after 100,000 iterations, showed that only 7 out of 100 attempts successfully achieved the correct electron density, all within the first 10,000 iterations. Failed cases remained unresolved, even after 90,000 additional iterations, highlighting two key issues: the lack of global optimization strategy, which leads most calculations to stagnation or incorrect local solutions, and independent reconstructions that lack information sharing mechanisms, preventing complementary advantages and group evolution.

To address these limitations, we integrate genetic algorithms with this iterative framework, introducing population-level optimization while preserving the fundamental strengths of direct methods. Algorithm 1 illustrates the pseudocode. The implementation utilizes the MPI framework with 100 parallel threads independently reconstructing electron densities. In the main loop, after 100 standard dual-space iterations, genetic operations commence: all thread electron densities are gathered; fitness is calculated; and selection, crossover, and mutation operations are performed to produce a new generation. The electron densities of the new generation are then distributed back to the threads for continued iteration. The algorithm employs an elite preservation strategy to ensure the best individuals directly enter the next generation. It includes mechanisms to prevent premature convergence by maintaining population diversity. The method carefully handles origin and symmetry relationships during density exchange, ensuring meaningful recombination of structural features.

The success of the direct method fundamentally relies on sufficient constraints from solvent regions. Only diffraction amplitudes can be measured while phases are lost. Since the lost phases contain approximately 50% of the structural information, successful phase recovery requires at least half of the electron density to be known or highly constrained. Since the solvent region has a constant density, theoretically, a solvent content exceeding 50% ensures that the number of unknown parameters (electron density values) does not surpass the number of experimental observations (diffraction amplitudes) [41,42,43]. However, practical applications typically require solvent content above 65% to compensate for experimental limitations including missing low-angle data, measurement uncertainties, and ambiguous protein–solvent boundaries.
**Algorithm 1** Pseudocode of the genetic algorithm-enhanced direct phasing method.  1:Initialize MPI with 100 ranks (CPU threads)  2:Initialize 100 random densities as initial population $P_{0}$;  3:Define the iteration counter iter:=0;  4:**while** termination criteria is not fulfilled **do**  5:   Modify densities by an iteration cycle of direct phasing method;  6:   **if** iter%100==0 && iter≠0 **then**  7:       Collect densities from all ranks to the root rank;  8:       **if** on the root rank **then**  9:          Align densities to the same origin choice of unit cell;10:          Compute $R_{work}$ and $R_{free}$ of each density;11:          Identify elite density by a sudden drop in $R_{work}$ and $R_{free}$;12:          **for** each elite density **do**13:              Directly inheritance to $P_{\mathrm{iter}+1}$. {Elite preservation};14:          **end for**15:          **for** each density in $P_{\mathrm{iter}}$ **do**16:              Compute the similarity metric *s*;17:              Compute $R_{mod}$ and the fitness $f_{mod}$ to prevent premature;18:              Compute the probability of selection $p_{mod}$;19:              **repeat**20:                  Randomly select a density in $P_{\mathrm{iter}}$ and generate a random number *r* from (0,1). {Selection operation};21:              **until** $r\leq p_{mod}$22:              Randomly select 10 segments of density to apply crossover operator. {Crossover operation};23:              Randomly select 1% grid points of density and replace their values by random numbers from (0,1). {Mutation operation};24:          **end for**25:       **end if**26:       Send the modified densities from the root rank to their original ranks;27:   **end if**28:   iter=iter+1;29:**end while**

Protein envelope determination relies heavily on weighted average density calculations [21]. In early iterations, the electron density appears chaotic and disconnected, making direct protein region identification challenging. The method employs a Gaussian-weighted average density ($w_{k,i}$) defined in Equation (1), where $\sigma_{0}$ controls the averaging radius. Initially, a larger $\sigma_{0}$ value (∼4.0 Å) accommodates noisy density distributions, gradually decreasing to ∼3.0 Å as the density improves. This weighted averaging, efficiently implemented through FFT-based convolution, enables reliable distinction between protein and solvent regions based on local density values and estimated solvent content.

(1) $$w_{k,i}=\sum_{j}^{N_{grid}}exp\left[-r_{ij}^{2}/\left(2\sigma_{0}^{2}\right)\right]g_{k,j}^{\prime }$$

where *k* represents the iteration number. *i* and *j* indicate cell grid points. $r_{ij}$ is the inter-grid distance, and $\sigma_{0}$ adjusts the weighted average radius (3.0∼4.0 Å). $g{’}_{k,j}$ represents the diffraction-constrained electron density at grid point *j*. The weighted average density and protein envelope evolve synchronously through iterations.

Different constraints are then applied to protein and solvent regions. Protein regions undergo histogram matching using reference structure statistics to enforce proper density distributions. Solvent regions are modified using the HIO algorithm, with negative feedback [11] defined in Equation (2). This dynamic relaxation mechanism helps prevent iteration stagnation while maintaining appropriate solvent characteristics. The protein envelope determination updates continuously during density evolution, ensuring consistent region identification throughout the reconstruction process.

(2) $$g_{k+1}=\left\{\begin{array}{c} g_{k}^{\prime }\;\;\;\;\mathrm{protein}\;\mathrm{region}\\ g_{k}-\beta g_{k}^{\prime }\;\;\mathrm{solvent}\;\mathrm{region} \end{array}\right.$$

where $g_{k}$ represents the initial electron density of the $k^{th}$ iteration and $g_{k}^{\prime }$ is the density after diffraction amplitude modification. The ${\left(k+1\right)}^{th}$ iteration density ($g_{k+1}$) blends the input ($g_{k}$) and output ($g_{k}^{\prime }$)—hence, the “hybrid” name. HIO applies negative feedback only to the solvent region, dynamically constraining its density to facilitate electron density evolution in the unit cell.

Progress monitoring employs multiple metrics throughout reconstruction. The diffraction data are divided into a 99% working set for the iterative phasing process (yielding $R_{work}$ in Equation (3)) and a 1% test set excluded from phasing (yielding $R_{free}$ in Equation (4)). $R_{work}$ reflects the deviation between the reconstructed electron density and experimental diffraction data, with lower values indicating closer proximity to the true structure. $R_{free}$ helps detect potential overfitting. For PDB-posted structures, mean phase error defined in Equation (5) relative to the posted structure offers additional validation.

(3) $$R_{work}=\frac{\sum_{h\in work}\left|\right|F_{obs}\left(h\right)|-\lambda |F_{cal}\left(h\right)\left|\right|}{\sum_{h\in work}\left|F_{obs}\left(h\right)\right|}$$

(4) $$R_{free}=\frac{\sum_{h\in free}\left|\right|F_{obs}\left(h\right)|-\lambda |F_{cal}\left(h\right)\left|\right|}{\sum_{h\in free}\left|F_{obs}\left(h\right)\right|}$$

(5) $$\Delta \varphi =\frac{\sum_{h\in work}arccos\left\{cos\left[\varphi_{true}\left(h\right)-\varphi_{cal}\left(h\right)\right]\right\}}{\sum_{h\in work}1}$$

where h represents the Miller indices, $|F_{obs}\left(h\right)|$ represents experimentally observed diffraction amplitudes, $|F_{cal}\left(h\right)|$ denotes amplitudes calculated from the reconstructed density, λ is a scale factor, $\varphi_{true}$ represents phases calculated from published PDB structures, and $\varphi_{cal}$ indicates phases from the reconstructed density.

A critical challenge in implementation is handling missing low-angle diffraction data blocked by the beam stop. These low-resolution data are crucial for protein envelope determination, necessitating their estimation through Equation (6). Given significant measurement uncertainties in the 100∼300 recorded very-low-resolution reflections, calculated amplitudes often replace experimental data below approximately 15 Å resolution, including those in the free dataset.

(6) $$|F_{miss}\left(h\right)|=\frac{\sum_{h\in work}\left|F_{obs}\left(h\right)\right|}{\sum_{h\in work}\left|F_{cal}\left(h\right)\right|}\left|F_{cal}\left(h\right)\right|$$

### 2.2. Detailed Implementation of Genetic Algorithm Components

#### 2.2.1. Population Initialization and Fitness Function

**Figure 2:**
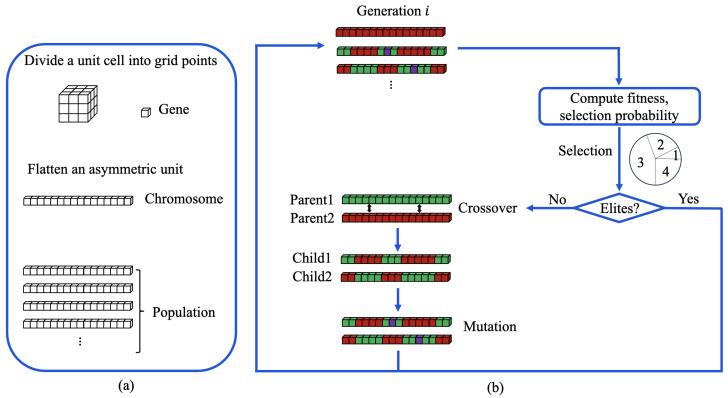
(**a**) A diagram of genetic algorithm components in protein crystallography. (**b**) Flowchart of genetic algorithm operations.

The integration of genetic algorithms with direct methods requires careful consideration of how to represent and manipulate electron density distributions within an evolutionary framework, as shown in Figure 2. The initial population is constructed by distributing the computation across 100 independent MPI processes. Each process starts with random electron density values and performs 100 iterations of standard dual-space calculations. The asymmetric unit is divided into uniform grid points, with electron density values at grid centers representing local density distributions. Electron density values at individual grid points serve as the fundamental genetic units, while the complete set of density values within an asymmetric unit forms a chromosome. This representation preserves spatial relationships while enabling genetic operations.

The fitness function plays a crucial role in guiding evolution toward optimal solutions [35]. We derived fitness values from $R_{work}$ in Equation (7), which can be used as an adaptive fitness function that accounts for both global optimization needs and local improvement potential:

(7) $$f^{i}=\left\{\begin{array}{cc} \frac{R_{thres}-R_{work}^{i}}{R_{thres}-R_{min}} & \mathrm{if}\;R_{work}^{i}<R_{thres}\\ 0 & \mathrm{if}\;R_{work}^{i}\geq R_{thres} \end{array}\right.$$

where $R_{thres}=R_{avg}+\left(R_{avg}-R_{min}\right)$ is used to accommodate the dynamic nature of reconstruction quality. This formulation ensures effective differentiation between solutions throughout the evolutionary process. The design of this fitness function reflects several key theoretical considerations. First, the threshold mechanism ($R_{thres}$) dynamically adapts to the population’s overall quality, providing appropriate selection pressure throughout different evolutionary stages. During early iterations when most solutions are far from optimal, a higher $R_{thres}$ allows more solutions to contribute to evolution, maintaining diversity. As solutions improve, $R_{thres}$ gradually decreases, focusing selection on higher-quality solutions. Secondly, by using ($R_{thres}-R_{work}^{i}$) rather than simply $1/R_{work}^{i}$, we create a more linear relationship between solution quality and fitness, preventing premature dominance of marginally better solutions. This linear scaling is particularly crucial during the middle stages of evolution, where small improvements in $R_{work}$ may represent significant structural refinements. Thirdly, normalizing by ($R_{thres}-R_{min}$) ensures comparable fitness scales across different iterations, maintaining consistent selection pressure throughout the evolutionary process.

#### 2.2.2. Selection, Crossover, and Mutation

Selection employs a roulette wheel mechanism where each solution’s probability of being chosen as a parent equals $p^{i}$, as defined in Equation (8).

(8) $$p^{i}=\frac{f^{i}}{\sum_{j=1}^{N_{recon}}f^{j}}$$

where $N_{recon}$ represents the total number of independent reconstructions ($N_{recon}=100$). This approach naturally balances exploration and exploitation by probabilistically favoring better solutions while maintaining opportunities for less optimal ones to contribute. After selection, genetic operations proceed with carefully optimized parameters derived from extensive testing. Details are shown in the Discussion section.

The crossover operation employs a multi-segment strategy optimized through systematic testing. Based on extensive experiments comparing different crossover ratios (40%, 45%, 50%, and 60%) and segment configurations (10, 25, and 50 segments), we determined that exchanging 50% of density values through 10 segments (each comprising 5% of grid points) provides optimal results. This approach preserves local density features while enabling effective information exchange between individuals.

Mutation operations involve random perturbations to maintain population diversity and escape local optima. Our studies of different mutation rates (1%, 3%, and 5%) revealed that modifying 1% of grid points with random density values between 0 and 1.0 achieves the best balance between diversity maintenance and convergence efficiency.

#### 2.2.3. Elite Preservation and Premature Convergence Prevention

Elite preservation ensures the stability of superior solutions by directly inheriting top-performing individuals to the next generation. Elite identification primarily relies on dynamic $R_{work}$ characteristics, using a sliding window approach to monitor convergence instead of direct $R_{work}$ values, as $R_{work}$ can exhibit significant fluctuations during iteration, even when the solution is improving overall. Sliding window averaging effectively filters out these short-term oscillations, providing a more reliable indicator of genuine convergence trends. We define $\Delta {\bar{R}}_{work}^{i}$ at iteration *i* as the difference between two time-window averages of $R_{work}$ values: ${\bar{R}}_{work,2}$ (the mean value over iterations (i−300) to (i−200)) minus ${\bar{R}}_{work,1}$ (the mean value over iterations (i−100) to *i*). This sliding window approach enables real-time monitoring of convergence behavior by comparing $R_{work}$ trends across different time scales. When $\Delta {\bar{R}}_{work}^{i}$ exceeds a threshold, empirically set to 0.02, indicating significant improvement such as a sudden drop in $R_{work}$, the corresponding electron density is designated as elite. The sliding window average of $R_{free}$ can also be used to evaluate elites.

**Figure 3:**
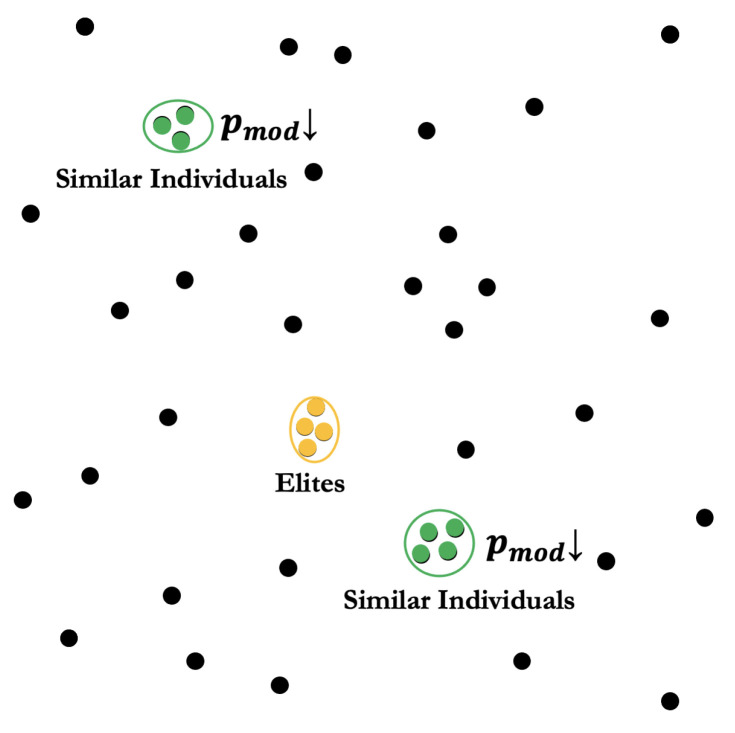
A diagram of elite preservation and premature convergence prevention. Each point represents an individual in a population. The selection probability of similar individuals should be reduced.

To address the premature convergence shown in Figure 3, a critical challenge in genetic algorithms, we developed a similarity-based selection probability adjustment mechanism. The similarity between any two electron density distributions is quantified through root-mean-square deviation, as shown in Equation (9).

(9) $$d^{ij}=\sqrt{\frac{1}{N_{grid}}\sum_{q=1}^{N_{grid}}{\left[g_{q}^{i}-g_{q}^{j}\right]}^{2}}$$

where $g_{q}^{i}$ represents the electron density of the $i^{th}$ individual at the $q^{th}$ grid point, where $N_{grid}$ denotes the total number of grid points in the asymmetric unit. When two electron density distributions are similar, the similarity measure ($d^{ij}$) approaches 0. Based on this, we define a convergence score ($s^{i}$) measuring the $i^{th}$ individual’s similarity to other population members, as shown in Equation (10).

(10) $$s^{i}=\sum_{j=1}^{N_{recon}}exp\left[-\frac{{d^{ij}}^{2}}{2\sigma^{2}}\right]$$

where σ represents the standard deviation of *d*. This Gaussian kernel-based function naturally weights closer solutions more heavily than distant ones. This mechanism is used to modify $R_{work}$ values as shown in Equation (11).

(11) $$R_{mod}^{i}=R_{thres}-\frac{R_{thres}-R_{work}^{i}}{s^{i}}$$

Similar individuals have larger convergence scores (*s*, where s>1 considering $d_{ii}=0$), which, in turn, leads to a larger $R_{mod}$, causing the fitness (*f*) to decrease and consequently reducing their probability of being selected.

The similarity-based selection probability adjustment mechanism uses root-mean-square deviation to quantify the diversity of electron density distributions, a choice based on both theoretical and practical considerations. Root-mean-square deviation provides a comprehensive measure of structural differences across all grid points, capturing both local and global variations in electron density distributions. Furthermore, by incorporating Gaussian kernel functions in the convergence score (*s*), we establish a continuous and differentiable measure of population diversity that naturally weights closer solutions more heavily than distant ones. This approach creates a smooth fitness landscape that effectively balances exploitation of promising solutions with exploration of the search space. When multiple solutions exhibit similar electron density patterns, their high convergence scores lead to reduced selection probabilities, thereby maintaining population diversity without sacrificing the quality of evolved solutions.

#### 2.2.4. Unit Cell Alignment and MPI Implementation

The unit cell density alignment procedure addresses the challenge of equivalent density representations arising from different origin choices, enantiomers, or density inversions in independent reconstructions. Our approach first establishes a reference frame by computing the weighted-average electron density to define protein regions (mask) based on estimated solvent content. Among all reconstructed densities, the one exhibiting the highest fitness serves as the reference standard for alignment. Each remaining electron density distribution then undergoes systematic evaluation of possible origin choices through mask comparison with this reference. The optimal origin choice is determined by maximizing the match between the candidate density’s protein mask and the reference mask. Final alignment is achieved through appropriate phase adjustments or density adjustments, either by translation or inversion, ensuring all density distributions share a consistent representation before genetic operations. This unified representation is crucial for meaningful density exchange during crossover operations, although the original origin choices are restored after genetic operations to maintain consistency in individual thread calculations.

The MPI implementation distributes computation across parallel processes [44], with genetic operations performed every 100 iterations. This interval balances the need for sufficient independent evolution through traditional dual-space iterations with timely information sharing across the population. Excessive frequency of genetic operations could disrupt the local optimization process, while excessive sparsity of operations would delay the propagation of beneficial features. Data gathering and distribution use MPI_GATHER and MPI_BCAST operations, respectively, with unit cell alignment carefully handled during these transfers to ensure meaningful density exchange during crossover operations. Testing on our Dell R740 workstation (52-core Intel Xeon processors at 2.1 GHz, 128 GB memory) demonstrated efficient parallel processing while maintaining algorithmic stability. Additional testing on multi-core Intel PCs showed similar results, although performance decreases when reconstruction counts exceed available threads. The complete implementation is available on Github [45], enabling broader application of this enhanced direct method approach.

## 3. Results

### 3.1. Systematic Validation and Performance Analysis

This section systematically examines the effectiveness of our genetic algorithm-enhanced direct method across diverse protein structures. From the Protein Data Bank, we selected 15 protein structures exhibiting high-quality diffraction data (published $R_{work}$ below 0.25) to comprehensively evaluate our method’s capabilities. Our test structures fall into three categories selected to systematically evaluate different aspects of the enhanced method: (1) Coiled-coil proteins with high solvent content (>65%), such as 6c4y, where traditional direct methods show moderate success rates but could be improved [27]. These proteins were selected because they represent challenging targets for both structure prediction methods (AlphaFold3 sometimes struggles with long helices or numerous short helices) and are frequently encountered in artificially designed protein structures [46]. (2) High-resolution structures (1.35∼1.97 Å) with medium solvent content (55∼65%), such as 4iqk. These structures challenge traditional direct methods despite their high resolution, making them ideal test cases for evaluating our method’s ability to handle lower-solvent-content scenarios. (3) Structures exhibiting NCS, including 6eik and 4uot. These proteins were chosen to test our method’s capability in handling local symmetry relationships and low solvent content (<55%).

**Figure 4:**
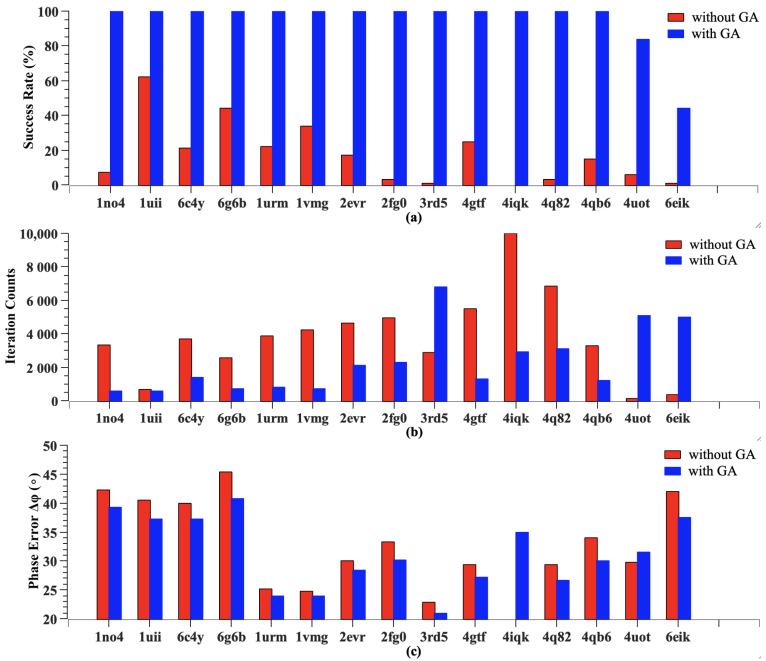
Comparison of (**a**) success rates, (**b**) iteration counts, and (**c**) phase error before and after algorithm improvement on 15 protein structures.

Figure 4 presents comparative analyses of success rates, iteration counts, and phase errors across all test cases. Each structure underwent 100 independent reconstruction attempts, starting from random initial values, with a maximum iteration limit of 10,000. The results demonstrate significant improvements in three key metrics: overall success rate, average iteration counts, and mean phase errors.

As shown in Figure 4a, the overall success rate increased from below 30% with traditional methods to nearly 100% with our enhanced approach. Among the test structures, 1uii shows an unusually high success rate (62%), even with traditional methods, owing to its high-quality diffraction data (2.0 Å resolution, $R_{work}$ = 0.216) and well-defined solvent regions that facilitate accurate envelope determination. In contrast, 2fg0, 3rd5, 4iqk, and 4q82 exhibit close-to-zero success rates with traditional methods despite attempts over thousands of iterations, reflecting the inherent limitations of conventional approaches near the 65% solvent content threshold. Additionally, for structures with high symmetry (such as 4uot and 6eik), performance improvement was particularly remarkable. However, the success rate did not reach 100%, primarily because the total number of iterations was limited to no more than 10,000. If the number of iterations were to be increased, the success rate could theoretically reach 100%.

As shown in Figure 4b, average iteration counts, calculated from successfully converged reconstructions, decreased substantially, typically from 4000∼5000 to 1000∼2000 iterations. This acceleration in convergence translated to reduced computation time from several hours to less than one hour on our test platform. Conversely, 3rd5 demonstrates high iteration count variability due to its extremely low success rate (1%) with traditional methods, making its iteration statistics less reliable. Moreover, the iteration count for 4iqk is 10,000; this is because the traditional direct phasing method failed to solve 4iqk, even after 10,000 iterations. Therefore, at least 10,000 iterations are needed. However, structures with high symmetry (such as 4uot and 6eik) showed much slower convergence. This counter-intuitive behavior arose because the crossover operation of the genetic algorithm interfered with the HIO algorithm, leading to an increase in the number of iterations.

As shown in Figure 4c, phase errors consistently decreased by 2∼3° across all test cases, with the resulting electron density maps showing sufficient quality. This improvement in accuracy demonstrates the method’s ability to not only find solutions more reliably but also to achieve better final results. The improvement in phase errors exhibits interesting variations. High-solvent-content structures like coiled coils show consistent phase error reductions of 2.5∼3.0°, while medium-solvent-content structures demonstrate more modest improvements of 1.5∼2.0°. Notably, since the traditional direct method failed to solve 4iqk, according to Equation (5), its phase error was set to 90°. Additionally, the phase error of 4uot slightly increased because the genetic crossover operations can temporarily disrupt the local symmetry.

Table 1 summarizes the phasing results of 15 structures before and after genetic algorithm improvement. By employing 100 independent reconstructions with random initial phases across 100 computational threads, the detailed performance metrics reveal the significant improvements introduced by the GA-enhanced direct phasing approach. Starting from random initial phases, the method consistently achieved final phase errors of less than $40^{\circ }$, generating highly interpretable electron density maps suitable for automatic model building using ARP/wARP [47] or Phenix AutoBuild [48].

As shown in Table 1, these structures feature high-resolution diffraction data with good quality, which is evidenced by their low PDB-posted $R_{work}$ values. Notably, despite its relatively low solvent content (55.82%), 4qb6 achieves better-than-expected phase accuracy due to its exceptionally high resolution (1.35 Å) and well-defined solvent boundaries. However, for structures with solvent content below 65%, successful reconstruction still heavily depends on factors including diffraction resolution, data quality, and protein envelope connectivity. The data for structure 4iqk are missing in the table is due to the inability of the unimproved direct method to obtain an accurate electron density. For all structures, the final phase error was calculated by first averaging all converged electron densities; then computing the phases corresponding to the averaged electron density; and, finally, calculating the phase error according to Equation (5). While some structures in the table possess NCS, only the last two structures implemented NCS density averaging: 4uot with C5 symmetry and 6eik with C7 symmetry. These two structures did not achieve a 100% success rate, mainly because the genetic algorithm’s crossover operation disrupted the density continuity between adjacent iterations, thereby affecting the performance of the HIO algorithm. In these cases of incomplete convergence, with the maximum iteration count set to 10,000, the average iteration count for all converged results typically remained around 5000.

### 3.2. Case Studies of Enhanced Direct Method with Genetic Algorithm

#### 3.2.1. Application to Protein Crystals with High Solvent Content: A Case Study of 1no4

Structure 1no4, a pre-assembly scaffolding protein with a $P2_{1}2_{1}2$ space group (a = 114.24 Å, b = 114.164 Å, c = 60.297 Å) [49], serves as our first detailed case study. This α-helical protein contains 2398 non-hydrogen atoms in its asymmetric unit with 77.7% solvent content. Despite its high solvent content theoretically favoring direct methods, traditional approaches achieved limited success. For reconstruction, we carefully chose several key parameters. The solvent content estimate was set to 70%, intentionally lower than the actual 77.7%, to ensure complete protein region coverage. Weighted average density calculations employed a dynamic $\sigma_{0}$ value, decreasing linearly from 4.0 Å to 3.0 Å as reconstruction progressed. This gradual reduction balanced noise suppression in early stages with feature preservation later. Low-resolution data treatment proved helpful: we applied a 15 Å cutoff, replacing experimental data below this threshold with calculated values due to typical measurement uncertainties in this range. The histogram matching process utilized the 2.2 Å resolution structure 1rb6 as reference, chosen for its similar resolution, average temperature factor, and overall protein architecture.

**Figure 5:**
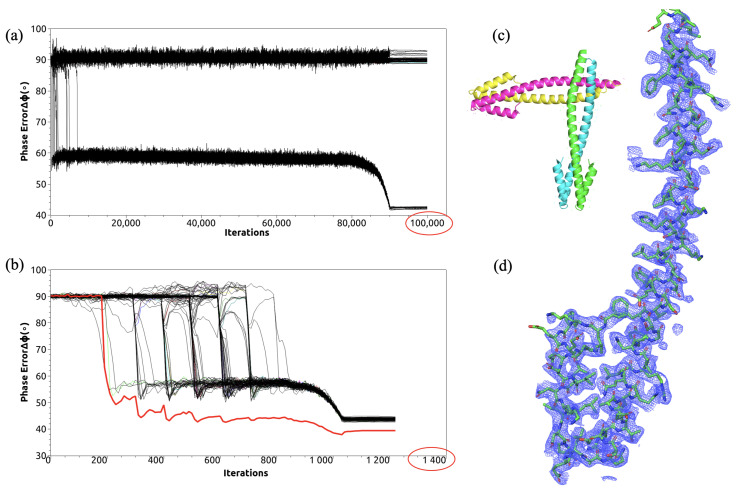
Comparison of the phasing performance before and after algorithm improvement on the structure of 1no4. (**a**) The evolution of phase errors in 100,000 iterations of the direct method. Only 7/100 independent reconstructions succeeded. (**b**) The evolution of phase errors in 1200 iterations of the genetic algorithm-enhanced direct method. All reconstructions succeeded. The red line represents the phase error corresponding to the average density of all successfully reconstructed densities. (**c**) PDB-posted structure of 1no4 shown in cartoon. (**d**) Reconstructed electron density of the genetic algorithm-enhanced direct method shown in blue mesh. The PDB-posted structure is superimposed as sticks.

Our enhanced method achieved remarkable performance improvements, as shown in Figure 5. In the traditional approach, only 7 out of 100 independent reconstructions succeeded, despite running 100,000 iterations each, with phase errors of 42.2°. The genetic algorithm enhancement achieved a 100% success rate within 1000 iterations, with computation time decreasing from several hours to under 30 min. By averaging the successful electron density reconstructions, phase errors were further reduced to 39.2°. Before the genetic algorithm was introduced, the direct phasing method struggled to reconstruct sufficient electron densities due to its low success rate, making it impossible to further minimize random errors through averaging. However, the genetic algorithm significantly improved the reconstruction success rate, enabling the averaging of all converged electron densities to maximize the reduction in random errors, ultimately decreasing the phase deviation by 3° in this case.

The implementation employed 100 parallel processes, each starting from random phases. Genetic algorithm operations began at iteration 100 and recurred every 100 iterations thereafter. The process monitored fitness values, selection probabilities, elite identification, crossover, and mutation operations continuously. Once all processes converged, as indicated by elite status across the population, the algorithm automatically transitioned to 200 cycles of solvent flattening. The final density averaging effectively reduced random errors by combining solutions that approached the global optimum from different directions in the high-dimensional search space.

**Figure 6:**
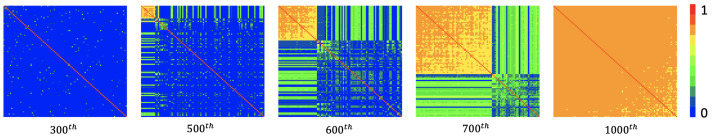
Performance of the premature prevention strategy in the GA-enhanced direct method on 1no4 at various iterations. Both axes of each frame show 100 independently reconstructed electron densities sorted by $R_{work}$ in ascending order. The heat map highlights strong correlation among successful reconstructions and diversity among others.

Premature convergence presents a significant challenge in genetic algorithms, potentially causing the population to lose diversity before reaching the global optimum. To validate our prevention strategy, we performed detailed analysis using structure 1no4. Figure 6 demonstrates the effectiveness of our similarity-based fitness adjustment mechanism. The convergence characteristics were monitored through correlation heat maps, where both axes represent 100 independently reconstructed electron densities arranged by increasing $R_{work}$ values, with colors indicating pairwise density correlations. At the 300th iteration, the heat map shows uniformly low correlations across all reconstructions, indicating high population diversity. By the 500th iteration, a small cluster of high correlations appears in the upper-left corner, representing the first few densities converging toward the global optimum, while the majority maintain their diversity. At the 1000th iteration, all reconstructions have successfully converged to the correct solution, as shown by uniformly high correlations. This progression confirms that our method maintains appropriate population diversity throughout the evolutionary process. Particularly noteworthy is the gradual expansion of the high-correlation region, indicating that beneficial features spread through the population without premature loss of diversity. The final averaged electron density from these diverse convergence paths achieves lower phase errors than individual solutions, demonstrating the additional value of maintained diversity throughout the reconstruction process.

This case demonstrates that population evolution mechanisms can effectively overcome the convergence barriers in traditional direct methods, even for structures with theoretically sufficient solvent content. There is one more thing that needs to be explained. Although the asymmetric unit within the unit cell of 1no4 contains four identical helices, these internal symmetry operations are challenging to reconstruct directly from the diffraction data. This is a frequently encountered scenario. In the next example, simple rotational internal symmetry is utilized to facilitate direct phasing.

#### 3.2.2. Application to Protein Crystals with Low Solvent Content and NCS: A Case Study of 6eik

**Figure 7:**
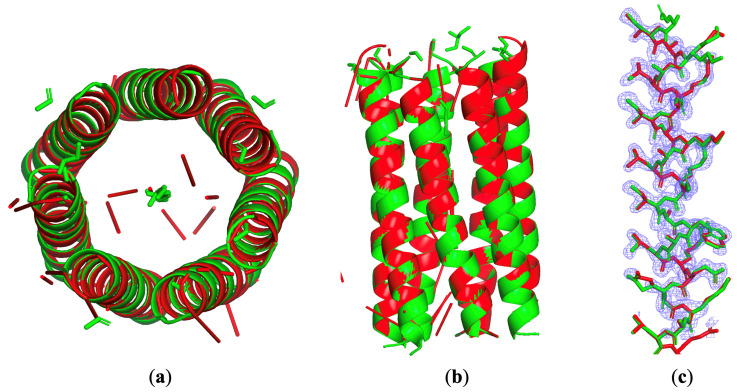
The PDB -posted structure of 6eik in green and the reconstructed model in red built directly from the reconstructed density. (**a**) Top view. (**b**) Side view. (**c**) Reconstructed density of the GA-enhanced direct method in blue mesh. The posted structure and the reconstructed model are superimposed.

Structure 6eik exemplifies the challenges and opportunities presented by NCS-containing structures with low solvent content. This engineered heptameric coiled-coil protein crystallized in space group $P4_{2}2_{1}2$ (a = b = 62.21 Å, c = 108.68 Å), with diffraction data extending from 1.52 Å to 40.78 Å at 99.1% completeness [50]. PDB-published $R_{work}$ was 0.161. The asymmetric unit contains 1826 non-hydrogen atoms with a notably low solvent content of 44.89%, placing it well below the theoretical limit for traditional direct methods. However, its seven-fold rotational symmetry (C7) provided the crucial additional constraints shown in Figure 7.

Implementation of NCS averaging required several specialized procedures [51,52,53]. If the protein structure is unknown, how can the NCS operations from be reconstructed from diffraction data? Using CCP4’s polarfn program [54], we analyzed self-rotation Patterson functions in a κ = 52° section to determine the initial NCS axis orientation [55]. The critical challenge lay in accurately positioning this axis within the asymmetric unit. In this example, we assumed the spatial orientation and position of the NCS rotation axis were known. We utilized a previously developed three-stage weighted density averaging approach with different radii: $w_{1}$ identified the NCS center, $w_{2}$ grew the NCS core region, and $w_{3}$ extended to the complete asymmetric unit. Identifying and refining NCS relationships from experimental data is time-consuming. Interested readers can refer to our previous paper [53]. At each step, weighted average density calculations employed specific σ values (15, 5, and 3 Å) optimized through systematic testing.

The reconstruction protocol integrated both genetic algorithm operations and NCS averaging. Each iteration involves first applying the HIO density modification, followed by NCS density averaging and histogram matching. Additionally, every 100 iterations, genetic modifications were applied. Finally, we performed density averaging over all successful reconstructions. Parameter optimization proved crucial: solvent content was set to 38%, deliberately underestimating to ensure robust protein region identification. The low-resolution cutoff at 15 Å addressed the typically larger errors in small-angle data. The $w_{3}$ weighted average density calculation used $\sigma_{3}$ = 3 Å. During histogram matching, the reference histogram came from 6g6e’s 1.52 Å resolution density distribution.

Results demonstrated dramatic improvement: success rates increased from below 1% to 44% across 100 independent trials. Each reconstruction consists of 10,000 iterations, with NCS density averaging applied every iteration and genetic algorithm operations every 100 iterations. The genetic algorithm proved essential for propagating successful features throughout the population. The significantly improved success rate stems from the NCS rotational symmetry operation, which accelerates the propagation of beneficial features when performing NCS density averaging. By increasing the iteration number beyond 10,000, a 100% success rate might be achieved. Additionally, the relatively high iteration count stems from the methodological conflict between the genetic algorithm crossover operation and the HIO density modification method. The HIO algorithm relies on maintaining electron density continuity between adjacent iterations, whereas crossover operations precisely disrupt this continuity. When NCS density averaging is introduced, crossover-generated density information rapidly spreads across the asymmetric unit via NCS symmetry operations, severing the inter-iteration electron density relationships that HIO depends on. Finally, the successful phasing of 6eik validates that genetic algorithm operations can enhance the effectiveness of NCS constraints, although the fundamental limitation of low solvent content cannot be completely overcome.

#### 3.2.3. Breaking Traditional Direct Method Limits: A Case Study of 4iqk

**Figure 8:**
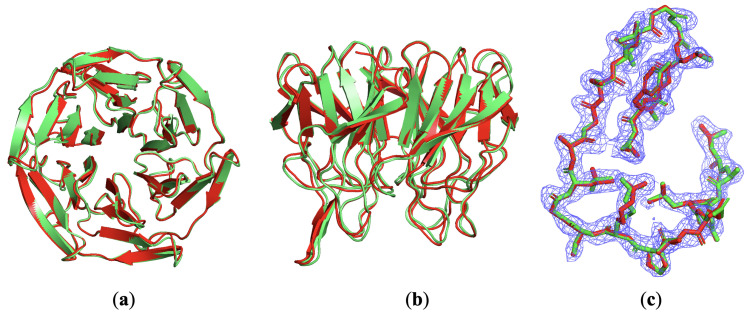
The PDB-posted structure of 4iqk in green and the reconstructed model in red built directly from the reconstructed density. (**a**) Top view. (**b**) Side view. (**c**) Reconstructed density of GA-enhanced direct method in blue mesh. The posted structure and the reconstructed model are superimposed.

4iqk represents a critical test of our method’s ability to solve structures near the theoretical limits of direct methods. Its structure is shown in Figure 8. This structure crystallized in space group C121 (a = 125.856 Å, b = 75.638 Å, c = 48.341 Å, β = 106.09°), with resolution spanning 1.97∼19.42 Å and 99.62% completeness [56]. The PDB $R_{work}$ is 0.153. The asymmetric unit contains 2341 non-hydrogen atoms, with a solvent content of 63.77%—remarkably close to the practical 65% threshold for traditional direct methods.

Direct methods rely fundamentally on solvent density constraints for phase recovery. The constant electron density in solvent regions provides crucial constraints through the HIO algorithm’s feedback mechanism. However, several practical factors complicate this theoretical foundation. Missing low-angle data from the beam stop, crystalline defects, and measurement errors typically necessitate solvent content above 65% for reliable reconstruction. Furthermore, reconstruction difficulty correlates strongly with protein region boundary complexity—a smoother boundary in the unit cell generally enables more accurate envelope determination. In implementing our enhanced method for 4iqk, we carefully optimized several parameters. Given the borderline solvent content, the weighted average density calculation proved particularly critical. We employed a gradually decreasing $\sigma_{0}$ value from 4.0 Å to 3.0 Å as reconstruction progressed to improve protein boundaries.

Traditional direct methods consistently failed to solve 4iqk, despite thousands of attempts, aligning with theoretical expectations for its solvent content. However, our enhanced method achieved convergence within an average of 2950 iterations across 100 independent trials. This success stemmed from the genetic algorithm’s ability to propagate partial solutions: when any process discovered favorable local density features, the selection and crossover mechanism rapidly shared these improvements across the population. The crossover operations proved particularly effective at combining successful features from different reconstructions, while the mutation rate of 1% provided sufficient variability to escape local optima without disrupting convergence. This borderline case establishes that the genetic algorithm-enhanced direct method can reliably solve structures near the theoretical solvent content limit where traditional approaches fail. The success with 4iqk suggests potential applications to other challenging structures previously considered unsuitable for direct methods. However, we emphasize that reconstruction success still depends heavily on data quality. Structures with poor diffraction data ($R_{work}$ > 0.3) remain challenging, even with our enhancements.

### 3.3. Comparison with Traditional Direct Methods and Previous GA Approaches

Our enhanced direct method represents a significant advancement over both traditional direct methods and previous genetic algorithm approaches in protein crystallography. Through systematic analysis of 15 test structures, we demonstrate several key improvements:

First, our method achieves significantly higher success rates compared to traditional direct methods. While conventional approaches typically achieve 10–30% success rates for structures with solvent content above 65%, our enhanced method consistently achieves 80–100% success rates across diverse test cases. This improvement is particularly notable for challenging structures like 4iqk (solvent content of 63.77%), for which traditional methods completely failed but our approach achieved consistent success.

Second, the integration of genetic algorithms with real-space density modification provides unique advantages over previous GA implementations. Earlier approaches focused primarily on phase optimization in reciprocal space, which might disrupt beneficial local density features. Our real-space approach better preserves and propagates locally optimized features while enabling effective global optimization through population evolution. The method’s ability to start from random phases and achieve ab initio structure determination represents a significant advance over previous GA methods that require initial phase estimates.

Third, our implementation demonstrates significant computational advantages. Through MPI parallelization and optimized genetic operations, we achieved convergence within 1–2 h on standard hardware, compared to several hours or failure with traditional methods. The parallel processing of tens of independent reconstructions enables automated error reduction through density averaging, improving phase accuracy by 2–3°. Our test cases demonstrate substantial improvements in key metrics: iteration counts reduced by approximately 50%, and computation time decreased from several hours to about one hour for non-NCS cases.

The method shows particular effectiveness for challenging scenarios including structures with solvent content near 65%, complex molecular boundaries, and non-crystallographic symmetry. However, certain limitations persist. Structures with solvent content below 65% remain challenging, and NCS reconstruction requires significant computational resources. For structures like 4uot and 6eik, determining NCS operators and boundaries from experimental data leads to longer convergence times. Users should note the increased computational requirements for NCS-containing structures due to the complexity of establishing correct NCS operators and boundaries. Additionally, the method’s effectiveness decreases with low-quality crystals or diffraction data (PDB $R_{work}∼$ 0.3 or resolution worse than 3.0 Å), as these conditions weaken the constant-density constraint of bulk solvent regions.

Despite these limitations, our method provides a valuable tool for structural biology by achieving reliable structure determination from random phases without requiring homology structures, predicted models, or experimental phasing.

## 4. Discussion

### 4.1. Optimization of Genetic Algorithm Parameters

**Figure 9:**
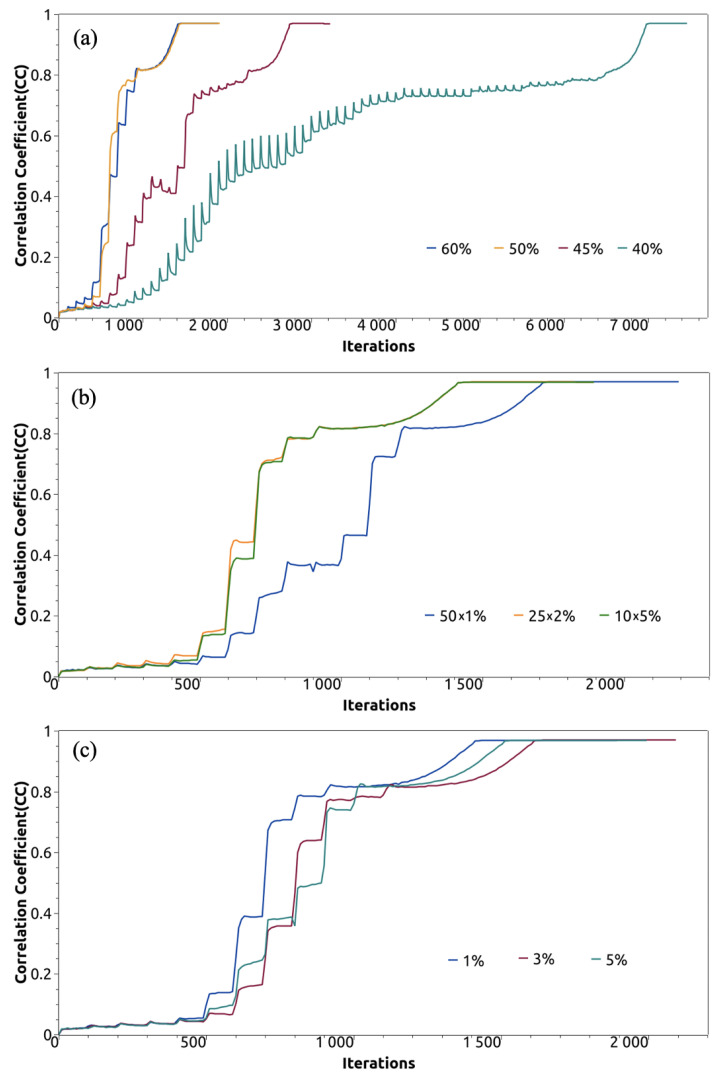
Comparison of the different (**a**) density exchange ratios, (**b**) density segments, and (**c**) mutation rates through correlation coefficients between reconstructed and true densities across iterations.

Our systematic investigation of genetic algorithm parameters revealed critical relationships between parameter choices and reconstruction performance. Extensive experiments comparing different density exchange ratios (40%, 45%, 50%, and 60%) demonstrated that exchanging 50% of density values provides optimal performance. Figure 9a illustrates that relationship through correlation coefficients between reconstructed and true densities across iterations, showing faster convergence with 50∼60% exchange compared to lower percentages. The similarity in performance between 50% and 60% exchange, combined with traditional genetic algorithm principles, led to our selection of 50% as the optimal value.

The number of segments for density exchange similarly proved crucial. Our comparison of 10 segments (5% each), 25 segments (2% each), and 50 segments (1% each), shown in Figure 9b, revealed that 10 segments achieved the fastest convergence. While 25 segments performed similarly, 50 segments significantly slowed convergence, likely due to excessive fragmentation of electron density features. This observation aligns with the spatial continuity requirements of protein electron density distributions.

Mutation rate optimization, depicted in Figure 9c, compared rates of 1%, 3%, and 5%. The results clearly demonstrate superior convergence with 1% mutation, while higher rates showed progressively slower convergence. This finding supports the theoretical expectation that minimal perturbation suffices for maintaining diversity while preserving beneficial features.

### 4.2. Technical Challenges and Solutions

Premature convergence posed the primary technical challenge in our implementation, requiring a sophisticated solution beyond standard genetic algorithm approaches. Our similarity-based fitness adjustment mechanism proved particularly effective by quantifying population diversity through root-mean-square density differences. The incorporation of Gaussian kernels in the convergence score provided a mathematically elegant way to weight similarity relationships, creating a continuous and differentiable measure of population diversity. When solutions exhibited high similarity, their inflated convergence scores automatically reduced their selection probabilities, effectively maintaining diversity without compromising evolutionary pressure toward optimal solutions.

The unified origin selection system addressed another fundamental challenge in crystallographic density manipulation. During genetic operations, equivalent density representations arising from different origin choices could potentially disrupt meaningful density exchange. Our solution involved temporarily unifying all density representations relative to a high-fitness reference before genetic operations, then restoring original origins afterward. This approach maintained both the integrity of density features during crossover and the computational consistency within individual threads.

### 4.3. Comparison with Existing Methods

Traditional applications of genetic algorithms in crystallography have primarily focused on phase determination for small molecules [35] and phase optimization with known initial phases [40]. These approaches either work only for high-resolution, low-complexity structures or require external phase information, fundamentally limited by insufficient utilization of protein crystal constraints and a lack of effective population evolution strategies. Existing methods provided valuable insights while revealing key challenges in conventional approaches, including defining effective fitness functions, handling medium- to low-resolution data, and dependence on initial phase estimates. Even with sophisticated genetic algorithms and density map characteristics like skewness and connectivity as fitness functions, achieving structure reconstruction from random phases remained challenging.

Our approach advances beyond these limitations in several aspects. First, we integrate genetic algorithms into a comprehensive direct phasing framework rather than limiting their use to phase optimization. Second, we exploit the constant density constraint of bulk solvent regions, which proves crucial for overcoming the phase problem. While existing methods highlighted difficulties in reconstructing protein structures from random phases, our method successfully addresses this challenge through innovative combination of genetic algorithms with real-space constraints, achieving ab initio determination of macromolecular structures. Third, compared to previous reciprocal-space phase optimization approaches, our real-space density modification strategy better preserves and propagates locally optimized features, as reciprocal-space modifications can disrupt beneficial local density patterns.

### 4.4. Current Limitations and Future Directions

Despite its successes, our method faces certain limitations. Structures with solvent content below 65% remain challenging, with success depending heavily on data quality, resolution, and protein envelope characteristics. Structures combining both rotational and translational NCS present particular difficulties for ab initio reconstruction. Looking forward, several promising directions emerge for method enhancement. The automation of NCS operator identification and refinement could benefit from advanced pattern recognition techniques, potentially incorporating local density correlation analysis to better identify symmetry relationships in noisy maps. Fitness function development could extend beyond global R-factors to incorporate local density features, particularly during early reconstruction stages where global metrics may not effectively distinguish partially correct solutions.

## 5. Conclusions

Our integration of MPI parallel genetic algorithms with the direct method has opened new possibilities in protein crystal structure determination. The key innovations and advantages of this method include the following:

First, we successfully implemented a population-based global optimization strategy in crystallographic phase determination. By simultaneously evolving multiple electron density reconstructions, our method effectively overcomes the limitation of local minima inherent in traditional direct methods. The adaptive fitness function and elite preservation mechanisms ensure robust convergence while maintaining population diversity.

Second, the integration of genetic algorithms with dual-space constraints provides a powerful framework for phase determination. The constant density constraint of solvent regions, combined with genetic operations, creates strong physical constraints that guide the evolution toward correct solutions. This synergy between classical crystallographic principles and evolutionary algorithms represents a significant methodological advancement in the field.

Third, our MPI parallel implementation makes the method practical for routine use. The efficient parallel architecture enables rapid exploration of the vast solution space, making previously intractable structures solvable within reasonable time frames. The automated workflow from random phases to interpretable electron density maps reduces the need for expert intervention in the phasing process.

However, there are important considerations regarding the method’s scope and limitations. The effectiveness strongly depends on solvent content and data quality—factors that affect the fundamental constraints available for phase determination. Additionally, while our method extends the applicability of direct methods to more challenging cases, it may not be suitable for all crystallographic problems, particularly those with very low solvent content or severe data quality issues.

Looking forward, several technical challenges remain to be addressed. These include improving the method’s performance with lower solvent content, enhancing the handling of complex NCS cases and optimizing computational efficiency for larger structural problems. Despite these challenges, our enhanced direct method provides a valuable alternative for solving novel protein structures when traditional approaches are insufficient. The success of this approach suggests that further integration of evolutionary algorithms with crystallographic methods could lead to additional breakthroughs in structure determination. As the method continues to evolve, it may help bridge the remaining gaps in our ability to solve challenging protein structures from experimental data.

## Figures and Tables

**Table 1:** Comparison of the reconstruction results before and after algorithm improvement.

						Success Rate (%)		Iteration Count		Final Δφ (°)	
PDB ID	Protein Name	Space Group	Resolution (Å)	PDB $R_{\mathrm{work}}$	Solvent Cont.(%)	Without GA	With GA	Without GA	With GA	Without GA	With GA
1no4	Viral scaffolding protein	$P2_{1}2_{1}2$	2.20	0.239	77.70	7	100	3325	600	42.2	39.3
1uii	Geminin coiled-coil domain	$P2_{1}2_{1}2_{1}$	2.00	0.216	65.30	62	100	670	600	40.5	37.3
6c4y	De novo two-helix protein	$P4_{3}22$	2.50	0.237	72.31	21	100	3694	1400	40.0	37.3
6g6b	De novo six-helix protein	$P4_{1}32$	2.30	0.216	74.99	44	100	2589	700	45.4	40.8
1urm	Peroxiredoxin	$P4_{1}2_{1}2$	1.70	0.146	63.50	22	100	3895	800	25.1	24.0
1vmg	Pyrophosphohydrolase	$I4_{1}22$	1.46	0.142	62.40	34	100	4253	700	24.7	24.0
2evr	Endopeptidase	$P4_{1}22$	1.60	0.159	61.16	17	100	4626	2100	30.1	28.4
2fg0	Endopeptidase	$P4_{1}2_{1}2$	1.79	0.154	63.86	3	100	4943	2300	33.3	30.2
3rd5	Putative uncharacterized protein	$P2_{1}2_{1}2_{1}$	1.50	0.133	65.00	1	100	2898	6800	22.8	21.0
4gtf	Thymidylate synthase	$I4_{1}22$	1.77	0.162	63.38	25	100	5511	1300	29.4	27.2
4iqk	Keap1 Kelch domain	C121	1.97	0.153	63.77	-	100	-	2950	-	35.0
4q82	Phospholipase	$P2_{1}2_{1}2$	1.85	0.153	62.49	3	100	6841	3100	29.3	26.7
4qb6	Sugar binding protein	$P2_{1}2_{1}2_{1}$	1.35	0.164	55.82	15	100	3302	1200	34.0	30.1
4uot	De novo five-helix protein	$C222_{1}$	1.69	0.178	63.90	6	84	152	5100	29.8	31.6
6eik	De novo seven-helix protein	$P4_{2}2_{1}2$	1.52	0.161	44.89	1	44	346	5000	42.0	37.5

## Data Availability

The diffraction data were downloaded from the Protein Data Bank at https://www.rcsb.org (accessed on 15 December 2024).
